# Supplementary material for: Biological weed control to relieve millions from Ambrosia allergies in Europe
Source: Nat Commun. 2020 Apr 21;11:1745. doi: 10.1038/s41467-020-15586-1 (PMC7174423; doi:10.1038/s41467-020-15586-1)
Supplement: Supplementary file 1 — Supplementary Information [file 41467_2020_15586_MOESM1_ESM.pdf]

Supplementary Information

Biological weed control to relieve millions from *Ambrosia* allergies in Europe

Schaffner *et al.*

Supplementary Note 1: Data for *Ambrosia* pollen exposure map

In the context of seasonal pollen integrals or pollen exposure, we use the terms ‘ragweed’ or ‘*Ambrosia*’ instead of ’common ragweed’ or ’*A. artemisiifolia*’ since pollen monitoring programs do not distinguish among different *Ambrosia* species (for the occurrence of other *Ambrosia* species in the study region see Materials and Methods in the main paper). The data used for mapping ragweed pollen exposure in Europe originated from studies conducted in the frame of the EU COST action SMARTER over the main centres in Italy[1], France[2], Austria[3], the Pannonian Plain[4] and supplemented with data from 19 different countries obtained from Šikoparija et al.[5], Höflich et al.[6], Buters et al.[7] and Peternel et al.[8]. Each geographical location with daily ragweed pollen integrals (number of pollen per cubic meter) corresponds to a calibration point in the mapping, where the data point provides the overall ragweed exposure in that point. Additional calibration points outside the main ragweed regions were obtained from studies that provided complete pollen calendars during the selected period (calendar day 200-305) in order to strengthen the interpolation results near the common ragweed invasion fronts. The inclusion requirement was that the studies report full calendars (including potential non-identified pollen) thereby documenting low or no occurrence of *Ambrosia* pollen. Based on this criterion, we included three stations in Spain[9-11] and six in Turkey and Cyprus[12-17] (Fig. 1b).

Supplementary Note 2: Overall sensitisation rates among the European population and ragweed sensitisation rates among the sensitised European population

A review of the literature produced 50 geo-referenced data sets from 16 European countries providing sensitisation rates among the general population based on skin prick tests or circulating Immunoglobulin E (IgE) tests. The data were extracted from four studies: 27 locations by Bousquet et al.[18], six locations by Ackermann-Liebrich et al.[19], one location by Blomme et al.[20] and 16 locations by Newson et al.[21] (Fig. 2b; Supplementary Data 2).

The review on ragweed sensitisation rates among sensitised persons, based on ragweed specific skin prick tests and ragweed specific circulating IgE tests, provided 80 geographical areas within 22 European countries from 29 different national or international studies[2,8,19,21-47]. In addition, ragweed sensitisation rate among the sensitised population was calculated at 27 locations using tabular information in Bousquet et al.[18], providing 22 additional sites and 5 supplementing site data. The data from 22 French locations from the study by Thibaudon et al.[2] provided annual normalised values for each region based on data collected during 2005-2008 and did not include percentages similar to all the other studies. In our calculations we assumed a similar number of patients per department which may introduce small errors. Here we used mean values for the entire period and rescaled the normalised values to actual values by using data from the medical centres in regions of Montpellier, Paris and Grenoble given in Heinzerling et al.[29] and Bousquet et al.[18] as these regions are also found in Thibaudon et al.[2]. Each of the 102 locations was given geographical attributes corresponding to the central town in the region, choosing the geographical coordinates of the university hospital or another large central hospital if they were present in the region (Fig. 2c; Supplementary Data 2).

To obtain a complete coverage of sensitisation rates across Europe, we included studies using skin prick tests and circulating IgE tests either alone or in combination to obtain a complete coverage of sensitisation rates across Europe. While the two methods may differ in sensitivity and specificity, they usually provide comparable results and are used interchangeably in allergy research[48,49].

Supplementary Note 3: Estimation of health costs due to ragweed allergies

Average treatment costs per patient and costs related to work absenteeism were calculated from data provided in the final report of the EU project on 'Assessing and controlling the spread and the effects of common ragweed in Europe'[50] and from a database compiled by the 'Agence régionale de santé Auvergne-Rhône-Alpes' (www.auvergne-rhone-alpes.ars.sante.fr/) from the Rhône-Alpes region in south-eastern France. Bullock et al.[50] summarized information on treatment costs from nine European countries, based on information provided by national experts and the project team.

The costs per patient and year provided in Bullock et al.[50] vary considerably and the distribution of the costs is highly skewed. We, therefore, decided to use the median (Euro 565 per year), rather than the average costs (Euro 1,168 per year). Also, due to a lack of data from many of the countries included in our analysis, we decided to use a European-wide average, rather than country-specific cost estimates. Bullock et al.[50] based their estimation of the average European ragweed allergic person annual treatment costs on the costs for immunotherapy treatment in the UK, which amounted to approximately Euro 3,030 in 2011. Assuming that the costs for immunotherapy treatment are the total cost for medication per patient over 10 years of treating allergic reactions to *A. artemisiifolia* pollen exposure, this resulted in Euro 303 per patient per year. Based on the average annual treatment costs, Bullock et al.[50] estimated that the total medical costs of ragweed in Europe amount to Euro 721 million (Euro 237-1,537 million) and the socio-economic costs related to work absenteeism to Euro 178 million (Euro 67-1,480 million), which is approximately 25% of the medical costs and thus higher than the percentage used in our study.

We based the costs related to work absenteeism, relative to the medical costs, on information obtained from the Rhône-Alpes region in south-eastern France (www.auvergne-rhone-alpes.ars.sante.fr/). Work time lost amounted to an average of 18.5% of the annual amount of treatment costs for the period of 2010-2015 (Supplementary Data 6), resulting in combined treatment and lost work time costs of Euro 565 * 1.185 = Euro 670 per patient and year.

Recently, Linneberg et al.[51] conducted a systematic review of European studies on the costs associated with allergic respiratory diseases. They found that the median direct

(treatment) costs for seasonal allergic rhinitis caused by pollen from studies published over the last 25 years amounted to Euro 426 per patient (mean costs Euro 436) and median indirect costs, mainly due to absenteeism, to Euro 538 per patient (mean costs Euro 813). Hence, while the European-wide average direct costs per patient for seasonal allergic rhinitis are lower than our estimates for ragweed pollen-caused allergies, the indirect costs and the overall costs (Euro 964) are higher.

Supplementary Data 7 summarizes the medical cost estimates due to *A. artemisiifolia* before the arrival of *O. communa* and at the point in time when *O. communa* will have colonised its entire environmental niche in Europe. The cost estimates were obtained for each country by multiplying the combined treatment and lost work time costs (Euro 670) with the number of clinically relevant ragweed sensitised persons. The table reports both the uncorrected as well as the purchasing power parity (PPP)-adjusted medical costs. PPP-adjusted costs were obtained by weighting the treatment and lost work time costs at the country level using PPP-adjusted health expenditures per capita for 2015 from the Global Health Expenditure Database[52]. Although the PPP approach remains debatable[53], we adopted the PPP-adjusted cost estimates as they are more conservative.

It is well documented that the exposure to *A. artemisiifolia* and *Artemisia* spp. can lead to extensive IgE cross-reactivity and clinical symptoms[54]. Thus, theoretically, a change in *A. artemisiifolia* pollen integrals might not change the situation for a large proportion of weed-sensitised patients, as pollen originating from related species will trigger allergic symptoms also in the absence of *A. artemisiifolia* pollen. However, as shown for the Rhône-Alpes region in south-eastern France (Supplementary Fig. 4b) and for the Milano area in Northern Italy (Supplementary Fig. 6d), *A. artemisiifolia* pollen integrals in heavily invaded regions in southern Europe are far higher than the pollen integrals for *Artemisia* spp. Hence, a change in *A. artemisiifolia* pollen integrals in these regions is likely to result in a corresponding change in number of patients. This is also supported by the highly significant relationship between the *A. artemisiifolia* pollen integral and the number of patients in the 313 communities of the Rhône-Alpes region (Supplementary Fig. 5). While some anti-allergic/asthma medication purchased during the flowering season of ragweed were most likely used to treat other allergies, the highly significant relationship shown in Supplementary Fig. 5 reveals that the number of

patients in the communities of the Rhône-Alpes region is largely determined by the size of the local *A. artemisiifolia* pollen integral.

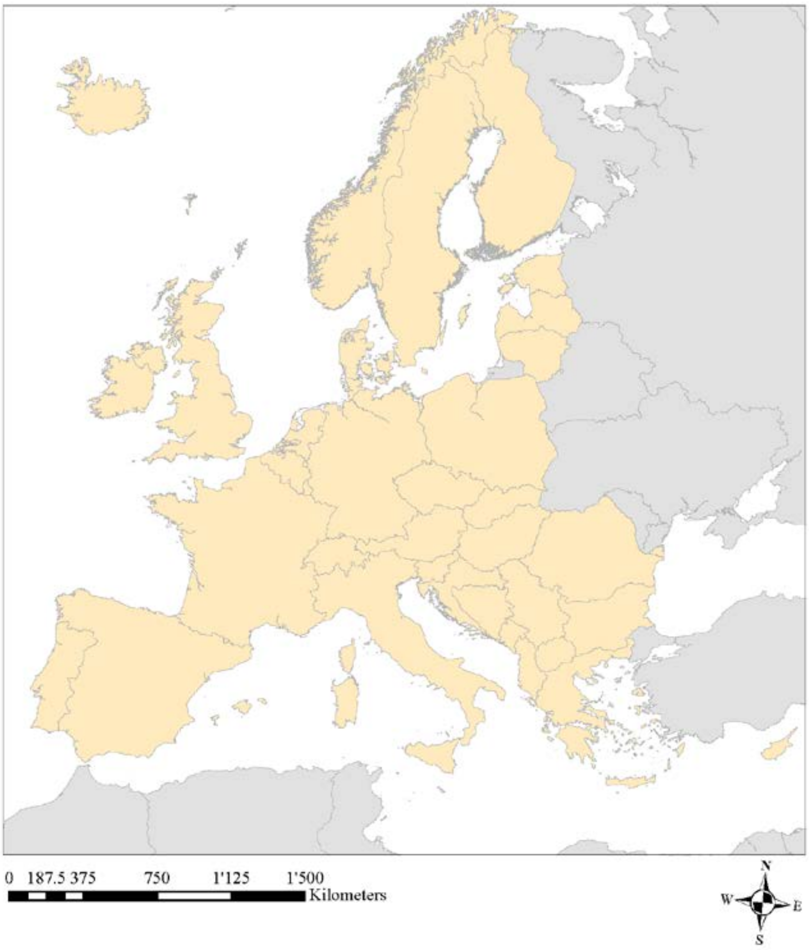

Supplementary Figure 1. Map of the study area.

The study area includes the EU countries, Albania, Bosnia-Herzegovina, Kosovo, Macedonia, Montenegro, Serbia, Switzerland, Norway and Iceland (countries coloured wheat).

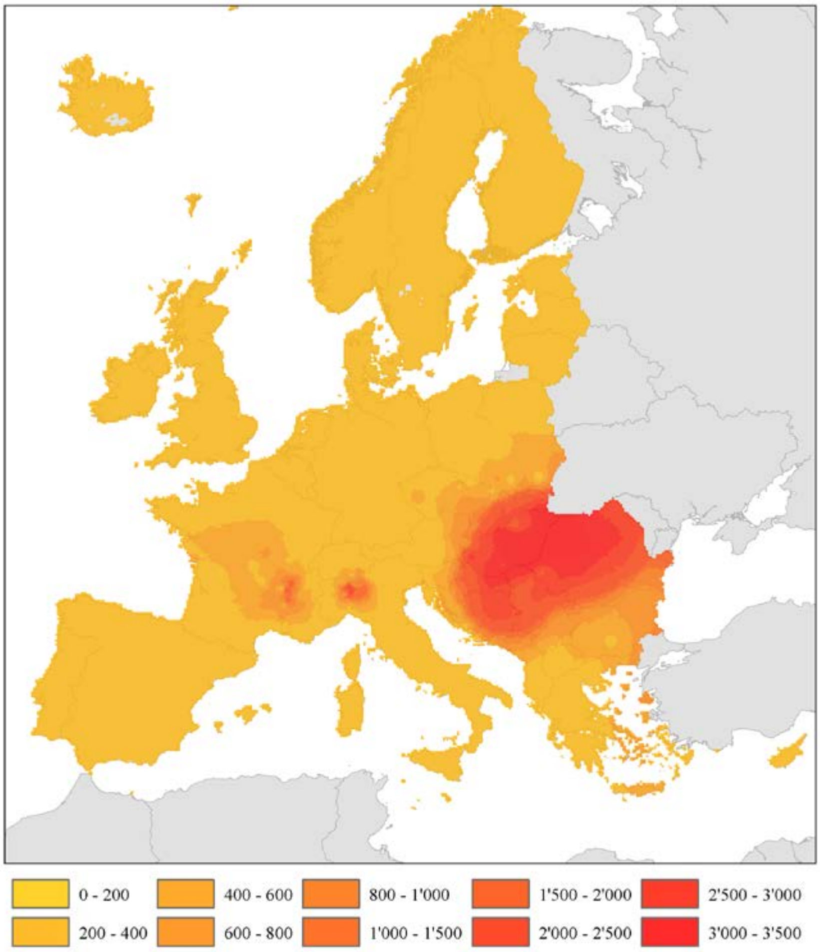

Supplementary Figure 2. Standard deviation of the interpolated seasonal pollen integrals for *Ambrosia* (number of grains per cubic meter of air) across Europe before the establishment of *Ophraella communa* (data from 2004-2012; see Fig. 1).

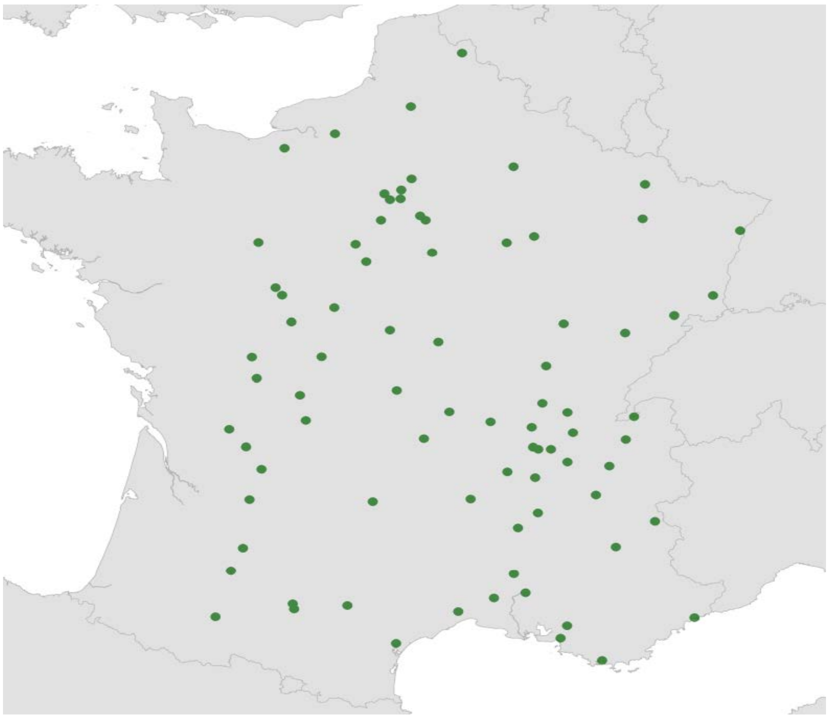

Supplementary Figure 3. Distribution of 62 pollen monitoring stations in France. Data on seasonal pollen integral for ragweed were used to interpolate the community-level pollen exposure in the Rhône-Alpes region.

a

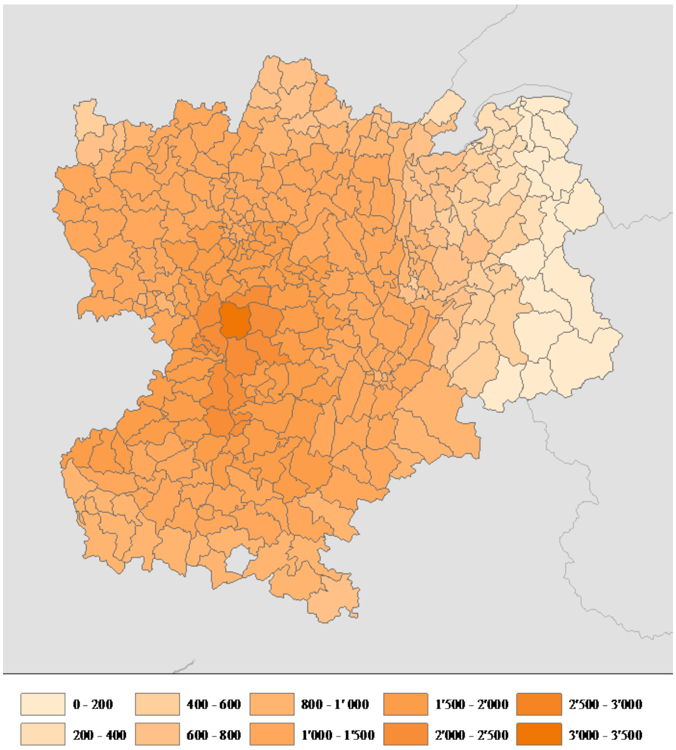
0 - 200
200 - 400
400 - 600
600 - 800
800 - 1'000
1'000 - 1'500
1'500 - 2'000
2'000 - 2'500
2'500 - 3'000
3'000 - 3'500

b

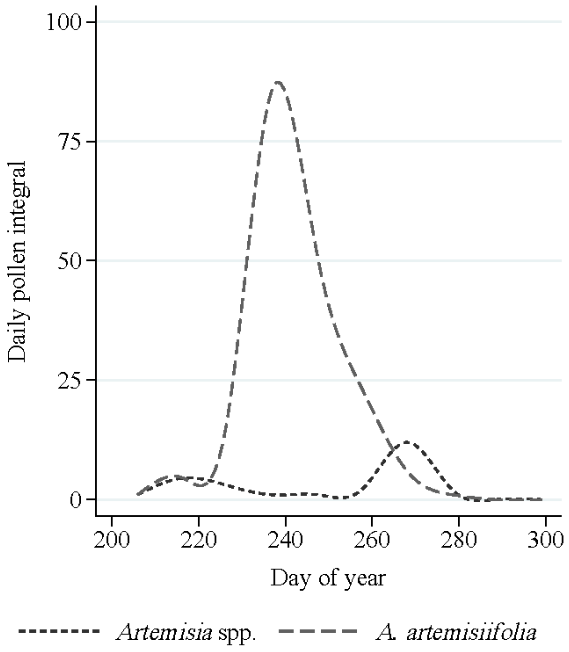
Daily pollen integral
100
75
50
25
0
200
220
240
260
280
300
Day of year
Artemisia spp.
A. artemisiifolia

Supplementary Figure 4. Seasonal pollen integrals for *Ambrosia* for the Rhône-Alpes region in France.

a Interpolation of seasonal pollen integrals for *Ambrosia* for the Rhône-Alpes region in France during the period 2007 to 2015. Given are average seasonal *Ambrosia* pollen integrals per community in the region, based on interpolation of seasonal pollen integrals obtained from all pollen monitoring stations in France (Supplementary Fig. 3).

b Comparison of daily pollen integrals for *Ambrosia* and *Artemisia* spp. during the flowering season in the Rhône-Alpes region in France (data from 2013-2018).

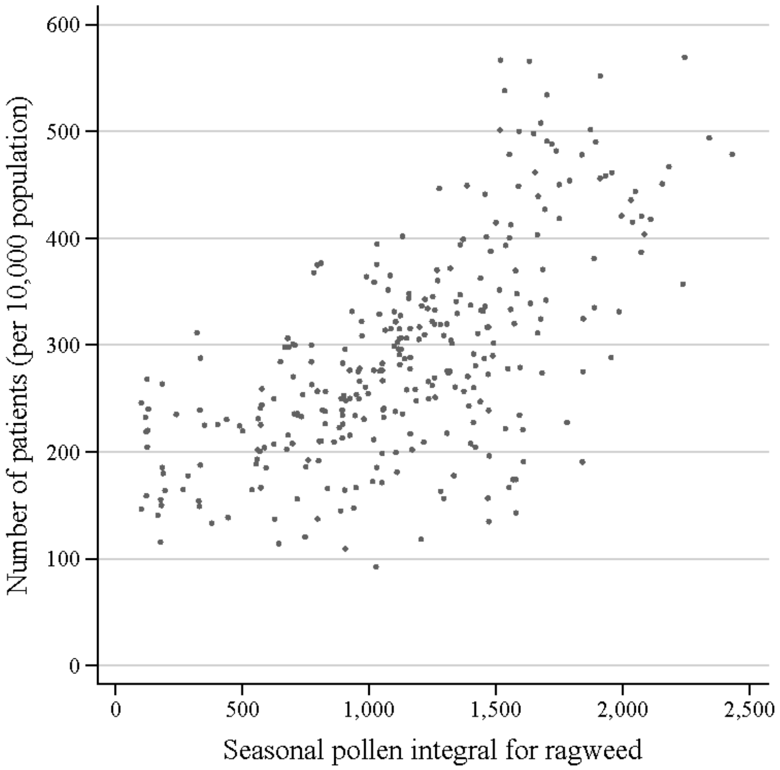

Supplementary Figure 5. Relationship between seasonal *Ambrosia* pollen integrals and the number of patients suffering from *A. artemisiifolia* allergies in the Rhône-Alpes region in south-eastern France.

The data are based on average seasonal *Ambrosia* pollen integrals for 313 communities in the region (period 2007 to 2015), cut-off at 3 pollen grains per cubic meter of air for calculation of season length to account for interpolation error. Regression: log(y) = 0,84 log(x), t = 87.84, P < 0.001.

a

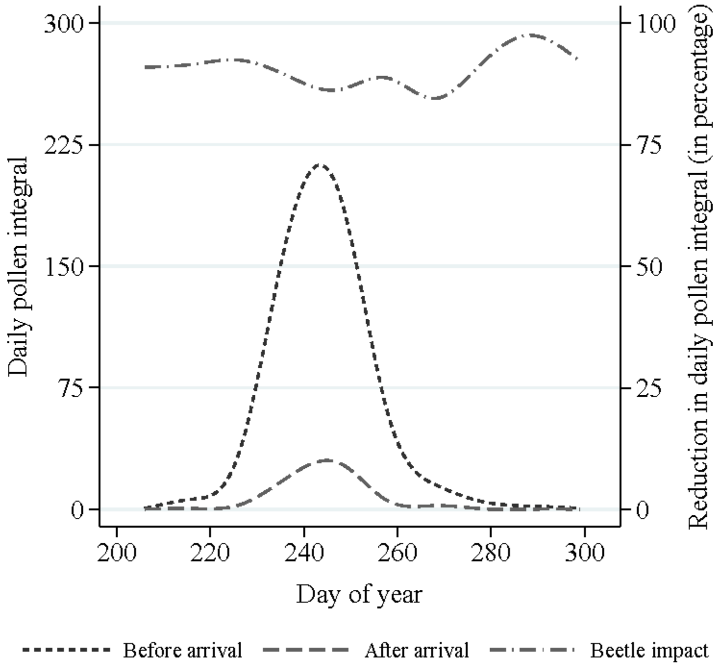
Daily pollen integral
Reduction in daily pollen integral (in percentage)
300
225
150
75
0
100
75
50
25
0
200
220
240
260
280
300
Day of year
Before arrival
After arrival
Beetle impact

b

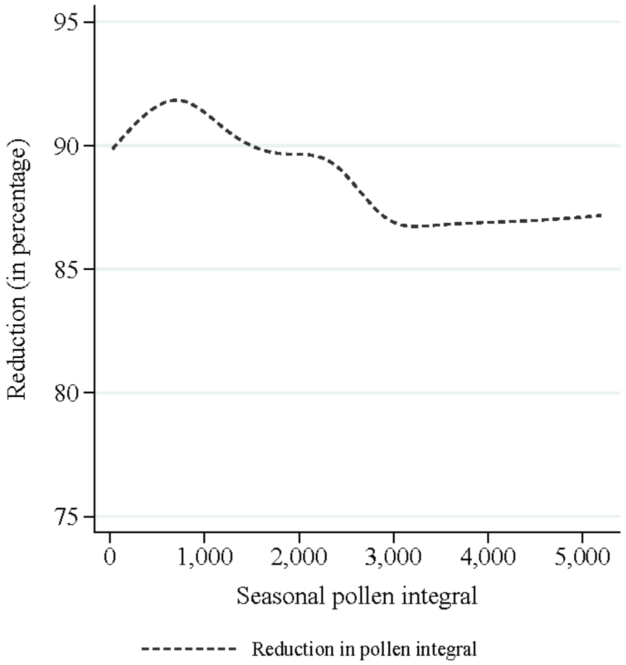
Reduction (in percentage)
95
90
85
80
75
0
1,000
2,000
3,000
4,000
5,000
Seasonal pollen integral
Reduction in pollen integral

166 **c**

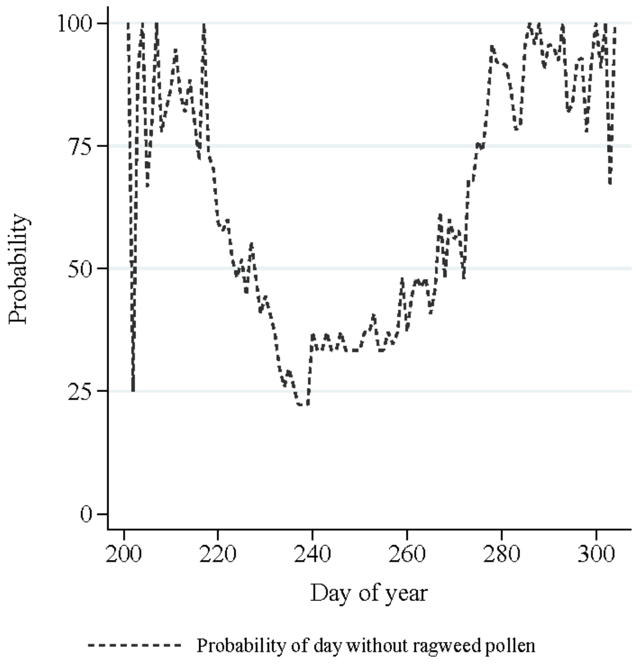

167

168 **d**

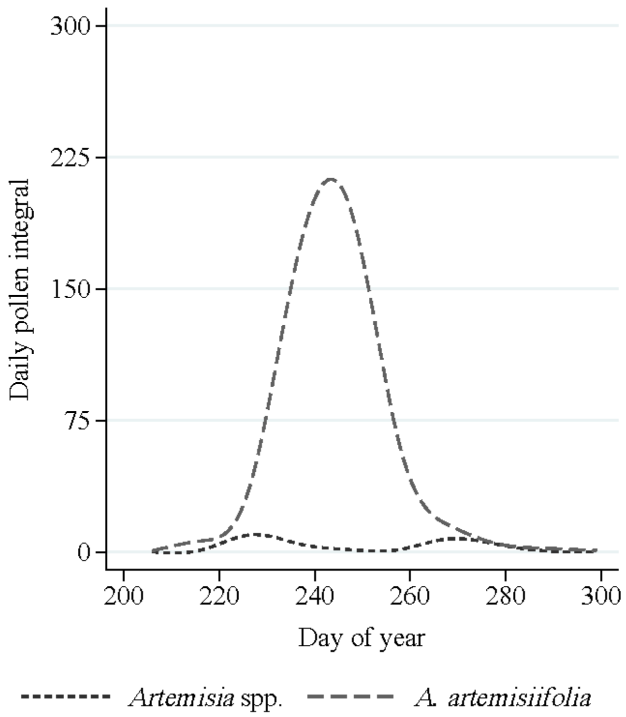

169

Supplementary Figure 6. Seasonal *Ambrosia* pollen integrals for the Milano area before and after the arrival of *Ophraella communa*.

a Average daily *Ambrosia* pollen integrals (number of pollen/m^3 and day) for the Milano area before the beetle's arrival (short-dashed line; 2004-2012) and after the beetle's arrival (long-dashed line; 2013-2018), and the beetle's relative impact on daily pollen counts (dash-dotted line).

b Estimated reduction in seasonal pollen counts due to the ragweed leaf beetle. The lines depict the cubic spline estimates using cross medians as knots to fit the spline function.

c Probability of days without *Ambrosia* pollen after the establishment of *O. communa*. Given is the period during which *Ambrosia* pollen were recorded prior to the establishment of *O. communa* (2004-2012).

d Average daily pollen counts for *Ambrosia* (long-dashed line) and *Artemisia* (short-dashed line) in the Milano area before the beetle's arrival (2004-2012).

a

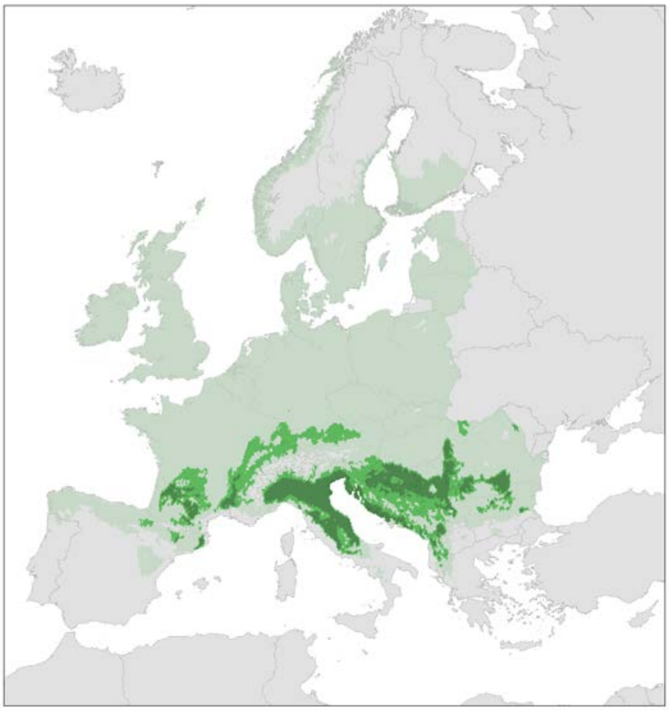

b

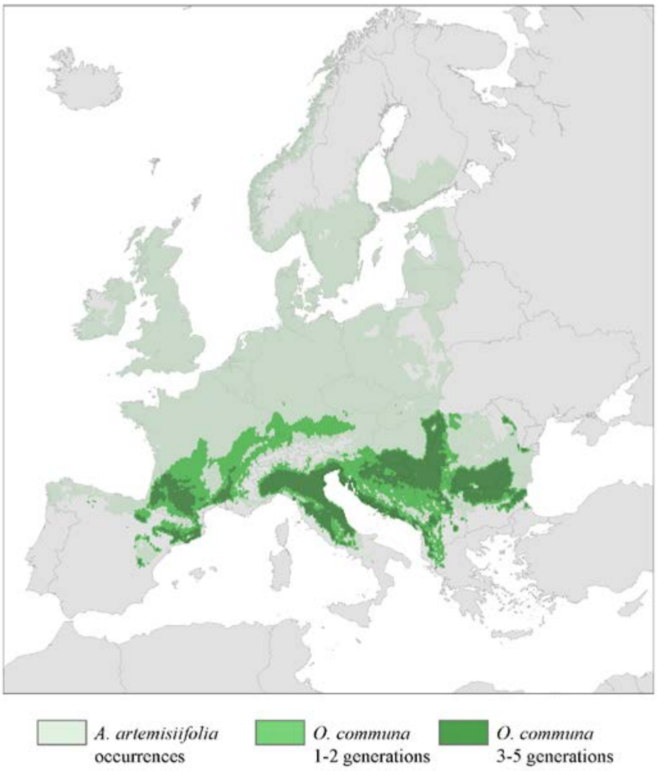
A. artemisiifolia occurrences
O. communa 1-2 generations
O. communa 3-5 generations

Supplementary Figure 7. Lower and upper limits of the combined 95% confidence intervals for the predicted distribution of *A. artemisiifolia* and number of generations per year of *Ophraella communa* in Europe.

a The lower limit of the 95% confidence interval for the predicted distribution of *O. communa* is shown with the upper limit of the 95% confidence interval for the species distribution model for *A. artemisiifolia*.

b The upper limit of the 95% confidence interval for the predicted distribution of *O. communa* with the lower limit of the 95% confidence interval for the species distribution model for *A. artemisiifolia*.

These two combinations represent the extreme range of the projected impact of *O. communa* on seasonal pollen integrals of *A. artemisiifolia* across Europe, relative to the CIs of the two species distribution models.

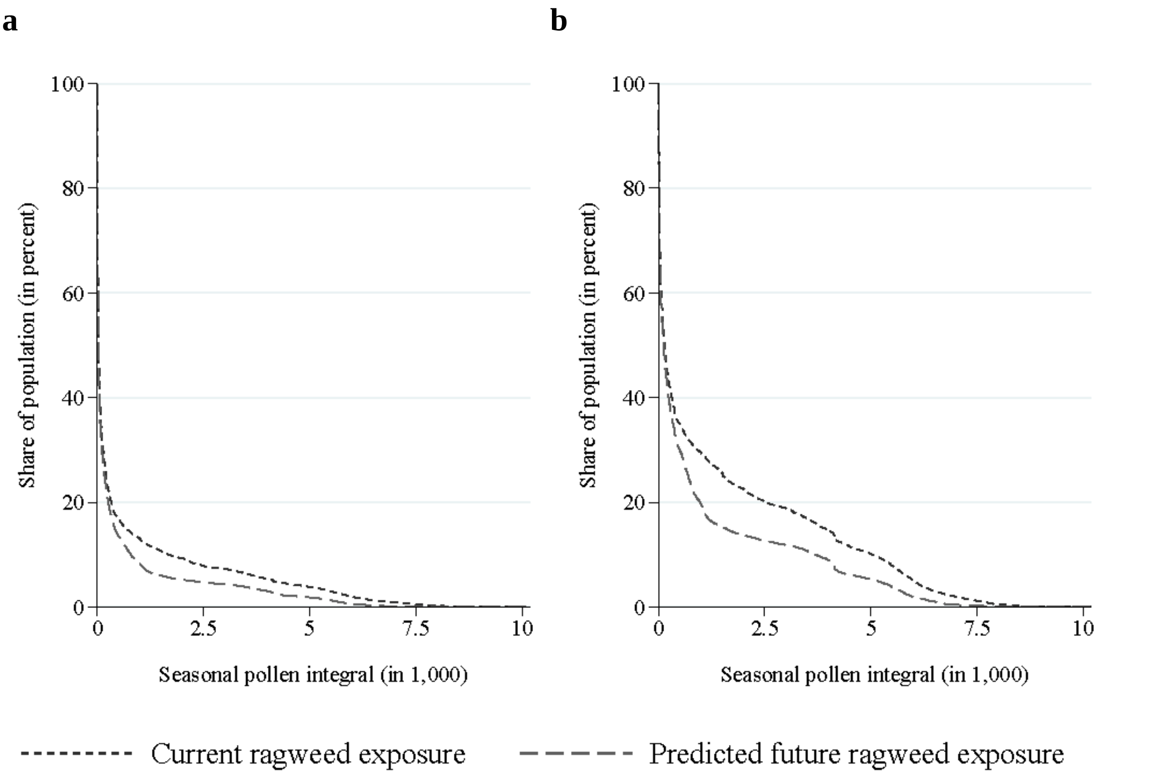

Supplementary Figure 8. Projected impact of the ragweed leaf beetle *Ophraella communa* on the seasonal exposure of the European population to *Ambrosia* pollen.

a Exposure of the general population.

b Exposure of the ragweed sensitised population.

Short-dashed line: exposure before the establishment of *O. communa* (2004-2012). Long-dashed line: projected exposure once *O. communa* will have colonised its entire environmental niche in Europe. The population share is defined as the share of people exposed to levels of common ragweed pollen above the pollen count threshold (10 pollen per cubic meter and season; see main text) divided by the total population.

Supplementary References

1. Bonini, M. *et al.* *Ambrosia* pollen source inventory for Italy: a multi-purpose tool to assess the impact of the ragweed leaf beetle (*Ophraella communa* LeSage) on populations of its host plant. *Int. J. Biometeo.* **62**, 597–608 (2017).
2. Thibaudon, M., Hamberger, C., Guilloux, L. & Massot, R. Ragweed pollen in France: origin, diffusion, exposure. *Eur. Ann. Allergy Clin. Immunol.* **43**, 209-215 (2010).
3. Karrer, G. et al. Ragweed (*Ambrosia*) pollen source inventory for Austria. *Sci. Tot. Environ.* **523**, 120-128 (2015).
4. Skjøth, C. A. *et al.* A method for producing airborne pollen source inventories: An example of *Ambrosia* (ragweed) on the Pannonian Plain. *Agric. For. Meteo.* **150**, 1203-1210 (2010).
5. Šikoparija, B. *et al.* Spatial and temporal variations in airborne Ambrosia pollen in Europe. *Aerobiol.* **33**, 181-189 (2016).
6. Höflich, C. *et al.* Potential health risk of allergenic pollen with climate change associated spreading capacity: Ragweed and olive sensitisation in two German federal states. *Int. J. Hyg. Environ. Health* **219**, 252-260 (2016).
7. Buters, J. et al. *Ambrosia artemisiifolia* (ragweed) in Germany – Current presence, allergological relevance and containment procedures. *Allerg. J. Int*. **24**, 108-120 (2015).
8. Peternel, R., Culig, J., Hrga, I., & Hercog, P. Airborne ragweed (*Ambrosia artemisiifolia* L.) pollen concentrations in Croatia, 2002–2004. *Aerobiol.* **22**: 161–168 (2006).
9. García-Mozo, H., Perez-Badia, R., Fernandez-Gonzalez, F. & Galán, C. Airborne pollen sampling in Toledo, central Spain. *Aerobiol.* **22**, 55-66 (2006).
10. Gonzalo-Garijo, M. A., Tormo-Molina, R., Muñoz-Rodríguez, A. F., Silva-Palacios, I. Differences in the spatial distribution of airborne pollen, concentrations at different urban locations within a city. *J. Investig. Allergol. Clin. Immunol.* **16**, 37-43 (2006).
11. Rodríguez-de la Cruz, D., Sánchez-Reyes, E., Dávila-González, I., Lorente-Toledano, F. & Sánchez-Sánchez, J. Airborne pollen calendar of Salamanca, Spain, 2000–2007. *Allergol. Immunopathol.* **38**, 307-12 (2010).

12. Gucel, S., Guvensen, A., Ozturk, M., & Celik, A. Analysis of airborne pollen fall in Nicosia (Cyprus). *Environ. Monit. Assess.* **185**, 157-169 (2013).
13. Tosunoglu, A. & Bicakci, A. Seasonal and intradiurnal variation of airborne pollen concentrations in Bodrum, SW Turkey. *Environ. Mon. Ass.* **187**, 167 (2015).
14. Kizilpinar, I., Dogan, C., Artac, H., Resli, I. & Pekcan, S. Pollen grains in the atmosphere of Konya (Turkey) and their relationship with meteorological factors in 2008. *Turk. J. Bot.* **36**, 344-357 (2012).
15. Altintas, D. U. *et al.* Relationship between pollen counts and weather variables in East-Mediterranean coast of Turkey, *Clin. & Dev. Immunol.* **11**, 87–96 (2004).
16. Tosunoglu, A. *et al.* Atmospheric pollen concentrations in Antalya, South Turkey. *Aerobiol.* **31**, 99-109 (2015).
17. Güvensen, A., Celik, A., Topuz, B. & Öztürk, M. Analysis of airborne pollen grains in Denizli. *Turk. J. Bot.* **37**, 74-84 (2013).
18. Bousquet, P. J. *et al.* Geographical variation in the prevalence of positive skin tests to environmental aeroallergens in the European Community Respiratory Health Survey I. *Allergy* **62**, 301-309 (2007).
19. Ackermann-Liebrich U. *et al.* Sensitisation to Ambrosia in Switzerland: a public health threat in waiting. *Swiss Med. Wkly.* **139**, 70-75 (2009).
20. Blomme, K. *et al.* Prevalence of allergic sensitisation versus allergic rhinitis symptoms in an unselected population. *Int. Arch. Allergy Immunol.* **160**, 200–207 (2013).
21. Newson, R.B. *et al.* Geographical variation in the prevalence of sensitisation to common aeroallergens in adults: the GA2LEN survey. *Allergy* **69**, 643–651 (2014).
22. Albertini, R. *et al.* Evolution of ragweed pollen concentrations, sensitisation and related allergic clinical symptoms in Parma (northern Italy). *Aerobiol.* **28**, 347-354 (2012).
23. Aleksic, A. & Aleksic, V. *Ambrosia* – Allergen being of more interest. Fourth Congress of the general Medicine Doctors of Serbia with International participation, Zlatibor, Serbia 19 – 22.09.2013 (2013).
24. Ariano, R. *et al.* Ragweed allergy: Pollen count and sensitisation and allergy prevalence in two Italian allergy centers. *Allergy Rhinol*. 6, 177–183 (2015).

25. Bottero, P., Vecchio, F. & Bonini, M. Allergies due à l'Ambroisie: les symptomes respiratoires. European Colloquium on *Ambrosia*, 29-30 March 2012, Lyon, France, p.31 (2012).
26. Cvetanov, V. *et al.* Allergic diseases in R. Macedonia. Institute of Occupational Health, WHO Collaborating Center and GA2LEN Collaborating Center & Macedonian Society of Basic and Clinical Immunology and Allergology, pp.261 (2006).
27. Cvitanovic, S., Znaor, L. & Kanceljak-Macan B. Allergic rhinitis and asthma in southern Croatia: impact of sensitisation to *Ambrosia elatior*. *Croatian Med. J.* **48**, 68-75 (2007).
28. De Weger, L.A. *et al. Ambrosia* in the Netherlands: Allergic sensitivities and spreading of plants and clumps. *Ned. Tijdschr. Geneeskd.* **153**, 798-803 (2009).
29. Heinzerling L. M. *et al.* GA2LEN skin test study I: GA'LEN harmonization of skin prick testing: Novel sensitisation patterns for inhalant allergens in Europe. *Allergy* **64**, 1498-1506 (2009)
30. Hemmer W., Schauer U., Trinca A., Neumann C. & Jarisch, R. Ragweed pollen allergy in Austria: a retrospective analysis of sensitisation rates from 1997 to 2007. *J. Allergy Clin. Immunol.* **127**, AB170 (2011).
31. Höflich, C. *et al.* Potential health risk of allergenic pollen with climate change associated spreading capacity: Ragweed and olive sensitization in two German federal states. *Int. J. Hyg. Env. Health* **219**, 252–260 (2016).
32. Ianovici, N., Panaitescu, C. B. & Brudiu, I. Analysis of airborne allergenic pollen spectrum for 2009 in Timişoara, Romania. *Aerobiol.* **29**, 95-111 (2013).
33. Kadocsa, E. & Juhász, M. Study of airborne pollen composition and allergen spectrum of hay fever patients in South Hungary (1990-1999). *Aerobiol.* **18**, 203-209 (2002).
34. Myszkowska, D., Leśkiewicz, K., Czarnobilska, E., Tokarska-Guzik, B. & Kasprzyk, I. The problem of ragweed pollen in Krakow against a background of other Polish regions and some allergological aspects. *Giorn. Eur. Aerobiol. Med. Amb. Infez. Aerotr.* **10**, 67 (2014).
35. Novakova, S., Novakova, P. & Yoncheva, M. Characteristics of sensitisation among adults with allergic rhinitis. *Sci. Technol.* **6**, 47-53 (2016).

36. Plaschke, P. *et al.* Skin prick tests and specific IgE in adults from three different areas of Sweden. *Allergy* **51**, 461–472 (1996).
37. Popescu, F. & Tudose, A.M. Ambrosia pollen sensitisation in allergic rhinitis patients from the central part of the Romanian Plain. *Rom. J. Rhinol.* **1**, 26-30 (2011).
38. Pozzi, P. L., Berra, D., Zanon, P., Chiodini, E. & Ortolani, V. Ragweed in Busto Arsizio: since 1986 to date. *Eur. J. Aerobiol. Environ. Med.* **10**, 75 (2014).
39. Ruëff, F. *et al.* Sensitisation to common ragweed in southern Bavaria: Clinical and geographical risk factors in atopic patients. *Int. Arch. Allergy. Immunol.* **159**, 65-74 (2012).
40. Rybníček, O., Novotná, B., Rybníčkova, E. & Rybníček, K. Ragweed in the Czech Republic. *Aerobiol.* **16**, 287-290 (2000).
41. Ščevková, J., Dušička, J., Hrubiško, M. & Mičieta, K. Influence of airborne pollen counts and length of pollen season length of selected allergenic plants on the concentration of sIgE antibodies on the population of Bratislava, Slovakia. *Ann. Agric. Environ. Med.* **22**, 451–455 (2015).
42. Testi, S. *et al.* Multicenter investigation to assess the prevalence of *Ambrosia* pollen allergy in Tuscany. *J. Investig. Allergol. Clin. Immunol.* **19**, 251-252 (2009).
43. Tosi, A., Wüthrich, B., Bonini, M. & Pietragalla-Köhler, B. Time lag between *Ambrosia* sensitisation and *Ambrosia* allergy: A 20-year study (1989-2008) in Legnano, northern Italy. *Swiss Med. Wkly.* **141**, 132-153 (2011).
44. Toth, I., Peternel, R., Gajnik, D. & Vojniković, B. Micro-regional hypersensitivity variations to inhalant allergens in the city of Zagreb and Zagreb county. *Coll. Antropol.* **35**, 31-37 (2011).
45. Večenaj, A. *et al.* Risk for de novo sensitisation to ragweed in Croatian children. *Eur. J. Aerobiol. Environ. Med.* **10**, 41 (2014).
46. Yankova, R., Zlatev, V., Baltadjieva, D., Mustakov, T. & Mustakov, B. Quantitative dynamics of *Ambrosia* pollen grains in Bulgaria. *Aerobiol.* **16**, 299–301 (2000).
47. Zvezdin, B. *et al.* Ambrosia pollen-a causative agent of allergic diseases of the respiratory tract. *Pneumon* **41**, 9-16 (2004).
48. Lake, I.R. *et al.* Climate change and future pollen allergy in Europe. *Environ. Health Persp.* **125**, 385-391 (2017).

49. Chauveau, A. *et al.* Skin prick tests and specific IgE in 10-year-old children: Agreement and association with allergic diseases. *Allergy* **72**, 1365-1373 (2017).
50. Bullock, J. *et al.* Assessing and controlling the spread and the effects of common ragweed in Europe (ENV.B2/ETU/2010/0037). European Commission, Final Report (2012).
51. Linneberg, A. *et al*. Burden of allergic respiratory disease: a systematic review. *Clin. Mol. Allergy* **14**, 12 (2016).
52. World Health Organization. Global Health Expenditure Database. Available at: https://apps.who.int/nha/database (accessed 8 December 2020).
53. Taylor, A. M. & Taylor, M. P. The purchasing power parity debate. *J. Econ. Pers.* **18**, 135–158 (2004).
54. Asero, R. *et al.* Concomitant sensitization to ragweed and mugwort pollen: who is who in clinical allergy? *Ann. Allergy Asthma Immunol.* **113**, 307-313 (2014).
55. Burbach, G.J. *et al.* GA^2LEN skin test study II: clinical relevance of inhalant allergen sensitizations in Europe. *Allergy* **64**, 1507–1515 (2009).
