# Supplementary material for: Biological weed control to relieve millions from Ambrosia allergies in Europe
Source: Nat Commun. 2020 Apr 21;11:1745. doi: 10.1038/s41467-020-15586-1 (PMC7174423; doi:10.1038/s41467-020-15586-1)
Supplement: Supplementary file 2 — Description Additional Supplementary Information [file 41467_2020_15586_MOESM2_ESM.pdf]

Description of Additional Supplementary Files

Biological weed control to relieve millions from *Ambrosia* allergies in Europe

Schaffner *et al.*

File Name: Supplementary Data 1

Description: Predicted *Ambrosia* pollen exposure before and after establishment and spread of *Ophraella communa*. Given are the mean exposure (seasonal pollen integral) and the corresponding 95 % confidence intervals at the country level for the time before the establishment of *O. communa* (2004-2012) and projected exposure once the beetle will have colonized its entire environmental niche in Europe. The confidence interval for the exposure before the establishment of *O. communa* accounts for the prediction error in the mean exposure and the confidence interval for the exposure after the establishment of *O. communa* accounts for the joint confidence intervals of mean exposure as well as ragweed and beetle niche prediction.

File Name: Supplementary Data 2

Description: Studies on sensitisation rates among general population and ragweed sensitisation rates among sensitised people used to map ragweed sensitisation rates in Europe.

File Name: Supplementary Data 3

Description: Estimated exposure to common ragweed pollen within the general and the ragweed sensitised population before and after arrival of *O. communa*. Point estimates and the corresponding 95% confidence intervals are provided for mean population and the 1%, 25%, 50%, 75% and 99% percentiles of population distribution.

File Name: Supplementary Data 4

Description: Number of ragweed sensitised persons (in 1,000) at the country level. Given are data on the general population (Center for International Earth Science Information Network 2016) and estimates of the population sensitised, sensitised to ragweed pollen, and clinically relevant sensitised to ragweed pollen. Country level estimates of the proportion of ragweed sensitised individuals who will experience clinical symptoms when exposed to ragweed pollen (clinically relevant ragweed sensitisation) were taken from Burbach et al (2009).

File Name: Supplementary Data 5

Description: Number of ragweed sensitised persons (in 1,000) exposed to *Ambrosia* pollen before and after the arrival of the ragweed leaf beetle *O. communa*. The table presents estimates of the ragweed sensitised and the clinically relevant ragweed sensitised population which was exposed to *Ambrosia* pollen before the arrival of *O. communa* (before 2013) and which will be exposed to *Ambrosia* pollen once *O. communa* will have colonized its entire environmental niche. Country level estimates of the proportion of ragweed sensitised persons who will experience clinical symptoms were taken from Burbach et al.[15]. Numbers in brackets indicate 95% confidence interval for the exposure before the establishment of *O. communa* (accounting for the prediction error in the mean exposure) and the confidence interval for the exposure after the establishment of *O. communa* (accounting for the joint confidence intervals of mean exposure as well as of ragweed and beetle niche prediction).

File Name: Supplementary Data 6

Description: Costs (in Euro 1,000) for medical care and goods and for work time losses in the Rhône-Alpes region due to ragweed allergies. Source: 'Agence régionale de santé Auvergne-Rhône-Alpes' (www.auvergne-rhone-alpes.ars.sante.fr/). The data are based on the consumption of medical care and medical goods by persons affiliated to the general health insurance scheme and cover the period 2010-2015 (period with data for all costs available). The treatment costs include costs of anti-allergy and anti-asthma (treatments of asthma crisis) medication, reimbursed during the ragweed flowering period; consultations by generalist or specialist doctors linked to the prescription of anti-allergy medications during the ragweed flowering period; annual costs for Immunoglobulin E (IgE) tests and skin prick tests; and annual costs for desensitisation (including immunotherapies). Socio-economic costs (absence from work) are based on per diems for sick leave of fewer than twenty days when linked to the prescription of anti-allergic drugs during the ragweed flowering period.

File Name: Supplementary Data 7

Description: Estimated medical costs (in Euro 1,000) due to seasonal common ragweed pollen allergy in Europe before and after the arrival of the ragweed leaf beetle *Ophraella communa*. The table provides the estimated costs at the country level before the arrival of *O. communa* (before 2013) and at the point in time when *O. communa* will have colonized its entire environmental niche in Europe. The table reports medical costs, based on the combined treatment and lost work time costs (Euro 670; Supplementary Note 3) multiplied by the number of clinically relevant ragweed sensitised persons, and purchasing power parity (PPP)-adjusted medical costs. Confidence intervals are provided in brackets (see also Supplementary Data 5).

File Name: Supplementary Data 8

Description: Duration of a generation of *O. communa* in a field study in Italy along a climatic gradient. Given are measures of growing degree days (GDD) > 13.3° C for the period from the egg stage to adult emergence of *O. communa*. Individual measures are based on a field study where two cohorts of five individually covered common ragweed plants each were inoculated with freshly laid *O. communa* eggs, set up in field cages along an environmental gradient in the southern Alps and monitored until emergence of the next generation’s adults.
