# Supplementary material for: Biological weed control to relieve millions from Ambrosia allergies in Europe
Source: Nat Commun. 2020 Apr 21;11:1745. doi: 10.1038/s41467-020-15586-1 (PMC7174423; doi:10.1038/s41467-020-15586-1)
Supplement: Supplementary file 12 — Reporting Summary [file 41467_2020_15586_MOESM12_ESM.pdf]

nature research

Corresponding author(s): Urs Schaffner, NCOMMS-19-07132D

Last updated by author(s): Feb 8, 2020

Reporting Summary

Nature Research wishes to improve the reproducibility of the work that we publish. This form provides structure for consistency and transparency in reporting. For further information on Nature Research policies, see Authors & Referees and the Editorial Policy Checklist .

Statistics

For all statistical analyses, confirm that the following items are present in the figure legend, table legend, main text, or Methods section.

n/a	Confirmed	
☐	☒	The exact sample size (*n*) for each experimental group/condition, given as a discrete number and unit of measurement
☐	☒	A statement on whether measurements were taken from distinct samples or whether the same sample was measured repeatedly
☐	☒	The statistical test(s) used AND whether they are one- or two-sided<br>*Only common tests should be described solely by name; describe more complex techniques in the Methods section.*
☒	☐	A description of all covariates tested
☐	☒	A description of any assumptions or corrections, such as tests of normality and adjustment for multiple comparisons
☐	☒	A full description of the statistical parameters including central tendency (e.g. means) or other basic estimates (e.g. regression coefficient) AND variation (e.g. standard deviation) or associated estimates of uncertainty (e.g. confidence intervals)
☐	☒	For null hypothesis testing, the test statistic (e.g. *F*, *t*, *r*) with confidence intervals, effect sizes, degrees of freedom and *P* value noted<br>*Give P values as exact values whenever suitable.*
☒	☐	For Bayesian analysis, information on the choice of priors and Markov chain Monte Carlo settings
☒	☐	For hierarchical and complex designs, identification of the appropriate level for tests and full reporting of outcomes
☒	☐	Estimates of effect sizes (e.g. Cohen's *d*, Pearson's *r*), indicating how they were calculated

Our web collection on statistics for biologists contains articles on many of the points above.

Software and code

Policy information about availability of computer code

Data collection	Microsoft Excel 14.0; ArcGis 10.3;
Data analysis	R (R Core Team 2014); Stata 15; computer codes can be made available upon request.

For manuscripts utilizing custom algorithms or software that are central to the research but not yet described in published literature, software must be made available to editors/reviewers. We strongly encourage code deposition in a community repository (e.g. GitHub). See the Nature Research guidelines for submitting code & software for further information.

Data

Policy information about availability of data

All manuscripts must include a data availability statement. This statement should provide the following information, where applicable:

- Accession codes, unique identifiers, or web links for publicly available datasets
- A list of figures that have associated raw data
- A description of any restrictions on data availability

All data except those shown in Supplementary Table 6 have been transferred to the data repository at the University of Worcester; the link is https://eprints.worc.ac.uk/7124/. The password to open the files is: 123Ophra45. A short description of all data files is provided in the file 'inventory.txt' in the inventory folder.
The data that support the analysis of the percent persons suffering from ragweed allergies in the Rhône-Alpes region are available from the French Network of Aerobiological Monitoring (RNSA) but restrictions apply to the availability of these data, which were used under written agreement for the current study, and so are publicly not available. Data are however available from M. Thibaudon upon reasonable request and with permission of RNSA.

Field-specific reporting

Please select the one below that is the best fit for your research. If you are not sure, read the appropriate sections before making your selection.

☐ Life sciences ☐ Behavioural & social sciences ☒ Ecological, evolutionary & environmental sciences

For a reference copy of the document with all sections, see nature.com/documents/nr-reporting-summary-flat.pdf

Ecological, evolutionary & environmental sciences study design

All studies must disclose on these points even when the disclosure is negative.

Study description

The manuscript includes several interrelated studies. First, we mapped seasonal total ragweed pollen counts in Europe during the period 2004 to 2012, i.e. before the accidental introduction of the North American leaf beetle Ophraella communa, by interpolating data from 296 European pollen monitoring sites to a 10 x 10 km grid. We then we mapped ragweed sensitisation rates in Europe by combining published data on (a) the overall sensitisation rate among the general population, and (b) the ragweed sensitisation rates among the sensitised population. In the latter cases, we provide the number of studies (and references) that were used for mapping the sensitisation rates across Europe. By multiplying the interpolated ragweed sensitisation rates with the European population and the median health costs per patient (literature data) and weighting them at the country level using purchasing power parity (PPP)-adjusted health expenditures, we were able to calculate the number of patients suffering from ragweed induced allergies in Europe and the overall health costs. A unique data set from south-eastern France allowed us to validate our European-wide approach. Based on the observed impact of the biological control agent Ophraella communa since its establishment in Northern Italy in 2013 and by modelling the number of generations of O. communa across its suitable habitat range in Europe, we were able to project the impact of this biological control agent on the number of patients and on health costs once the beetle will have colonized its environmental niche in Europe.

Research sample

In all studies, we describe the sampling units and the number of samples included. In the impact experiments, insect exclosure treatment was achieved by alternatively spraying one of the three insecticides acetamiprid, deltamethrin and lambda-cyhalothrin. In the field study to assess the temperature-dependent development of O. communa, ambient temperature along the altitudinal range was used to calculate average growing degree days. The collection of occurrence data for A. artemisiifolia and O. communa for the species distribution models are described in detail in Appendix S2 in Sun et al. (2017, Ecosphere).

Sampling strategy

The data used for mapping ragweed pollen exposure in Europe originated from studies conducted in the frame of the EU COST action SMARTER over the main centres in Italy, France, Austria, and the Pannonian Plain and supplemented with data from 19 different countries. A review of the literature was conducted to collect geo-referenced data sets providing a) sensitisation rates among the general population and b) ragweed sensitisation rates among the sensitised persons.

Data collection

Ragweed pollen data recorded at the monitoring sites and literature data on sensitisation rates were collected by Carsten Skjøth, Maira Bonini and Letty de Weger. Suzanne Lommen collected the data on the impact of O. communa on ragweed pollen production, and Benno Augustinus collected the data on the temperature-dependent development rate of O. communa. Treatment costs per patient and costs related to work absenteeism were calculated from data provided in the final report of the EU project on 'Assessing and controlling the spread and the effects of common ragweed in Europe'16 and from a database compiled by the 'Agence régionale de santé Auvergne-Rhône-Alpes' (www.auvergne-rhone-alpes.ars.sante.fr/) from the Rhône-Alpes region in south-eastern France.

Timing and spatial scale

The European-wide pollen data were collected between 2004 and 2012 and the pollen data from Northern Italy between 2004 and 2018. The field impact study was conducted between 2014 and 2016 (data on pollen production collected in 2015 an 2016) and the field study to assess temperature-dependent development rate of O. communa in 2016 (first cohort started in late June/early July, second cohort in early August). The species distribution model for O. communa and A. artemisiifolia was completed in 2017.

Data exclusions

No data were excluded from the analysis.

Reproducibility

The field study on the impact of O. communa on pollen production was conducted over two years. The results in year 1 and year 2 were similar, suggesting reproducibility of the findings.

Randomization

The treatments in the impact study were randomized.

Blinding

Blinding was not used in our study. Blinding would have been impossible in the field work where the students were responsible for setting up and maintaining the experimental plots as well as for data collection.

Did the study involve field work? ☒ Yes ☐ No

Field work, collection and transport

Field conditions

Field studies on the impact of O. communa on ragweed pollen production and on temperature-dependent developmental rate of O. communa then allowed us to project the effect of this biological control agent, once it will have colonized its environmental niche, on the number of patients and on health costs in Europe.

Location

To assess the impact of O. communa on ragweed pollen production, we selected three sites in northern Italy where A. artemisiifolia and O. communa co-occurred: Corbetta (N45.4709 E8.9368), Magnago (N45.5707 E8.7855) and Grugliasco (N45.0654 E7.5923). Elevation is 140, 190 and 290 m asl, respectively. Average annual temperature in the region is 12.5-13°C

	and annual rainfall 850-1000mm.<br>The temperature-dependent developmental time of O. communa was studied along an altitudinal gradient in the Southern Alps, approximately 50 km north of the Milan area. The five field sites were set up on sun-exposed grasslands or in private gardens at 130 m (in the Po plain), 250 m, 480 m, 700 m and 1230 m asl, covering a temperature range of approximately 7°C.
Access and import/export	In all cases, authorisation to conduct field work was received by the landowners or communal authorities prior to setting up the experimental plots. Official requests for insecticide application in the experimental plots were submitted by the Local Health Authority, Milano Città Metropolitana, to the competent authorities of Corbetta, Magnago and Grugliasco. Permission for insecticide application was obtained from Magnago and Grugliasco, but not from Corbetta. Insecticides were only applied by trained persons wearing protective clothing.
Disturbance	At the highest elevation of the altitudinal gradient, A. artemisiifolia did not occur naturally. Care was taken to remove all inflorescences from the plants during the experiment to avoid seed set, and all plant material was removed and properly disposed at the end of the study.

Reporting for specific materials, systems and methods

We require information from authors about some types of materials, experimental systems and methods used in many studies. Here, indicate whether each material, system or method listed is relevant to your study. If you are not sure if a list item applies to your research, read the appropriate section before selecting a response.

Materials & experimental systems

n/a	Involved in the study
☒	☐ Antibodies
☒	☐ Eukaryotic cell lines
☒	☐ Palaeontology
☐	☒ Animals and other organisms
☒	☐ Human research participants
☒	☐ Clinical data

Methods

n/a	Involved in the study
☒	☐ ChIP-seq
☒	☐ Flow cytometry
☒	☐ MRI-based neuroimaging

Animals and other organisms

Policy information about studies involving animals; ARRIVE guidelines recommended for reporting animal research

Laboratory animals	The study did not involve laboratory animals
Wild animals	Pupae of O. communa were collected in the surroundings of the experimental sites. Freshly hatched females were mated and then allowed to oviposit on potted common ragweed plants. Once the experiment was completed, O. communa adults were again released.
Field-collected samples	Our manuscript does not include laboratory studies, so no animals were collected and maintained in culture.
Ethics oversight	No ethical approval was required.

Note that full information on the approval of the study protocol must also be provided in the manuscript.
